# Potential therapeutic use of relaxin in accelerating closure of cranial bone defects in mice

**DOI:** 10.14814/phy2.14106

**Published:** 2019-06-02

**Authors:** Kirk P. Conrad, Ean G. Phillips, Jessica Jiron, Julie Bailes, Biswadeep Dhar, YanPeng Diao, Jose Ignacio Aguirre, Joshua F. Yarrow

**Affiliations:** ^1^ Department of Physiology and Functional Genomics, Obstetrics and Gynecology University of Florida College of Medicine Gainesville Florida; ^2^ D.H. Barron Reproductive and Perinatal Biology Research Program University of Florida Gainesville Florida; ^3^ Research Service Malcom Randall VA Medical Center North Florida/South Georgia Veterans Health System Gainesville Florida; ^4^ Department of Physiological Sciences College of Veterinary Medicine University of Florida Gainesville Florida; ^5^ Division of Nephrology, Hypertension & Renal Transplantation Department of Medicine University of Florida College of Medicine Gainesville Florida; ^6^ Brain Rehabilitation Research Center Malcom Randall VA Medical Center North Florida/South Georgia Veterans Health System Gainesville Florida; ^7^ Division of Endocrinology, Diabetes, and Metabolism University of Florida College of Medicine Gainesville Florida

**Keywords:** Angiogenesis, blood flow, bone fracture, bone scaffold, calvaria, femur

## Abstract

Bone fractures are associated with considerable morbidity and increased mortality. A major limitation to healing is lack of bone blood flow, which is impaired by physical disruption of intraskeletal and/or periosteal vasculature by the fracture. Thus, pharmacological interventions are needed to improve osseous blood flow, thereby accelerating bone fracture closure. Relaxin is secreted by the ovary and circulates in rodents and humans during pregnancy. Because relaxin might benefit bone fracture healing by stimulating angiogenesis, vasculogenesis (and potentially osteogenesis) through mobilization and activation of bone marrow progenitor cells, and by increasing blood flow via vasodilation, we investigated whether relaxin administration would accelerate closure of a calvarial defect in mice. Whether administered systemically by osmotic pump or locally by collagen scaffolds for ~2 week period after lesioning, relaxin did not accelerate bone healing. Despite implementing relaxin doses that reached plasma concentrations spanning the physiological to supraphysiological range, testing the closure of two different sizes of calvarial lesions, allowing for different intervals of time from instigation of cranial lesion to euthanasia, and investigating mice of different ages, we did not observe a significant benefit of relaxin in bone lesion healing. Nor did we observe stimulation of blood vessel formation in the bone lesion by the hormone. An incidental finding was that relaxin appeared to enhance trabecular bone growth in an uninjured control bone (femur). Although the results of this study were not supportive of a therapeutic benefit for relaxin on calvarial defect closure, future investigation is needed employing different animal species and experimental models of bone fracture.

## Introduction

Bone fractures significantly increase the risk of mortality, and medical expenditures range from approximately $7000 to nearly $19,000 per fracture (depending on site), such that annual hospitalization costs exceed that of myocardial infarction, stroke, or breast cancer (Singer et al. 2015). These direct costs are compounded by indirect costs associated with absenteeism and short‐term disability. Moreover, the prevalence of osteoporosis is much higher in women than men as is fracture incidence (Singer et al. 2015). In the US Military, return‐to‐duty criteria necessitate full recovery from bone fracture for front‐line active duty service members, which can take upwards of several months. Unfortunately, US Military involvement in Operation Enduring Freedom/Operation Iraqi Freedom and other recent conflicts resulted in a high incidence of traumatic fractures for front‐line active duty service members (Wade et al. 2006; Lew et al. 2010). The incidence of traumatic fractures is expected to increase in future conflicts, in part due to the increasing usage of improvised explosive devices by adversaries. Consequently, failed or delayed bone healing is a major clinical problem in both the general population and the military, which necessitates the development of novel pharmacologic agents to accelerate bone healing.

A major rate‐limiting step in bone healing is the degree of osseous blood flow, which is impeded by concomitant physical disruption of the intraskeletal and/or periosteal vasculature following fracture. In addition, blood flow to the bone may be further compromised due to pre‐existing vascular disease in individuals who smoke, have metabolic syndrome, elevated cholesterol, or diabetes mellitus (Conte and Vale 2018). Angiogenesis (branching of existing blood vessels) is essential for bone fracture healing, and recent studies also reveal important roles for vasculogenesis (de novo formation of blood vessels) and osteogenesis both mediated by bone marrow‐derived progenitor cells (BMPC) (Matsumoto et al. 2006; Otsuru et al. 2008; Wang et al. 2011). Therefore, therapeutic agents are needed that will improve bone perfusion, and hence, healing of fractures.

**Figure 1:**
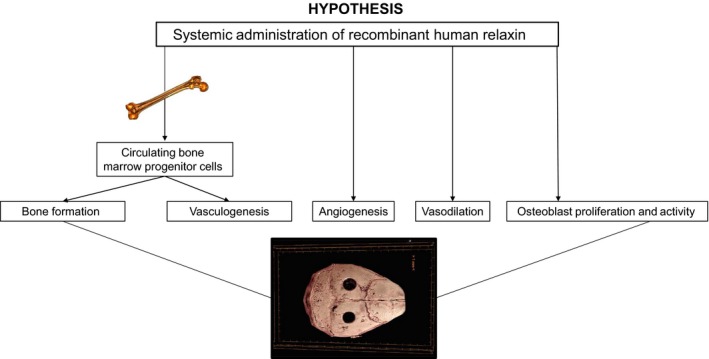
Bone perfusion is a rate‐limiting step in bone formation, and hence, healing. We hypothesized that, in a murine calvarial lesion model, administration of recombinant human relaxin would increase circulating bone marrow derived angio‐/osteogenic progenitor cells and enhance their uptake into the lesion site promoting vasculogenesis and bone formation, while concurrently stimulating angiogenesis, vasodilation, and bone formation by direct effects on osteoblasts. This study was not supportive of a beneficial role for recombinant human relaxin delivered either systemically or locally via collagen scaffolds applied to the lesions.

A logical approach to improve bone fracture healing would be to harness a therapeutic agent, which possesses the following attributes: (1) abets local angiogenesis, (2) stimulates vasculogenesis and osteogenesis by mobilizing and activating BMPC, (3) enhances blood flow via vasodilation, (4) directly stimulates bone formation, (5) counteracts inflammation, and (6) is clinically safe. To‐date, we are unaware of a single FDA‐approved agent that possesses all of these beneficial characteristics. However, as detailed next, the hormone relaxin fulfills these criteria, and thus may provide a novel approach to hasten bone fracture healing (Fig. 1).

Relaxin is a natural hormone in women secreted by the corpus luteum, which circulates in the luteal phase and during pregnancy (Sherwood 1994). Although it is unlikely to circulate in men, relaxin administration is nevertheless bioactive, leading to vasodilation, because males also have relaxin receptors in arteries (Danielson et al. 2000; Debrah et al. 2005; Novak et al. 2006; Jelinic et al. 2014; Ghosh et al. 2017). Several investigators reported that relaxin promotes angiogenesis in cycling and pregnant endometrium, skin wounds, muscle injury, and ischemic myocardium (reviewed in (Jeyabalan et al. 2007)). Relaxin increases human BMPC migration and nitric oxide production in vitro (Segal et al. 2012). Moreover, relaxin administration augments circulating BMPC, and enhances their incorporation and differentiation into endothelial cells in a matrigel plug assay in mice, that is, vasculogenesis (Segal et al. 2012). Circulating relaxin contributes to maternal vasodilation during pregnancy (at least in rats), and administration of relaxin to female and male, rodents and humans elicits renal and systemic vasodilation (Conrad 2011). Relaxin synergistically enhanced bone morphogenic protein 2‐induced osteoblast differentiation and bone formation in vitro suggesting that relaxin may enhance bone formation in vivo (Moon et al. 2014). A potential advantage of relaxin over other agents like granulocyte colony‐stimulating factor (G‐CSF) routinely used to mobilize BMPC is that relaxin may exert an anti‐inflammatory rather than inflammatory response (Masini et al. 2004; Brecht et al. 2011). In the case of G‐CSF, its inflammatory response leads to undesirable side‐effects (Fischmeister et al. 1999). Finally, based on several clinical trials in heart failure, recombinant human relaxin (rhRLX; Serelaxin) has a good safety profile (Said and Mukherjee 2013; Ghosh et al. 2017). In light of the aforementioned attributes, relaxin may be a novel therapeutic for improving bone fracture healing. The purpose of this work was to determine whether rhRLX administration accelerates bone healing of a calvarial defect model in mice.

## Materials and Methods

### Mice

In this proof‐of‐principle study, we investigated male mice, because men are currently the majority of front‐line soldiers. Power analysis was based on Wang and coworkers (Wang et al. 2011), in which they tested the role of AMD3100, a C‐X‐C chemokine receptor type 4‐antagonist to mobilize BMPC and accelerate closure of the same calvarial defect model as employed herein. These investigators observed significant differences using six treated and control mice each. We purchased C57Bl/6J male mice from Jackson Laboratory, and all animal procedures were approved by the University of Florida Institutional Animal Care and Use Committee, and the Animal Care and Use Review Office of the USAMRMC Office of Research Protection.

### Mouse experimental procedures

In Protocols 1–3, we used different rates of rhRLX infusion by subcutaneous osmotic pump (Alzet Osmotic Pumps, Durect, Co.), in order to span low, medium, and high concentrations in the blood. In Protocol 4, we employed a collagen scaffolding to deliver relaxin locally to the bone lesion. Protocol procedures are detailed below.

### Protocol 1 – recombinant human relaxin subcutaneous infusion: 1.0 *μ*g/h (Segal et al. 2012)

The general protocol was adapted from the study by Wang et al. (2011). Male mice of 13–15 weeks of age were anesthetized with isoflurane using a portable anesthesia machine (Summit Medical, Bend, OR). After shaving the head and aseptically preparing the surgical area, a mid‐line skin incision was made with a scalpel blade starting between the ears and ending over the occipital bone. The incision window was first positioned over the left and then right parietal bones, in order to make 1.5 mm bilateral calvarial lesions with a dental burr being careful not to injure the underlying dura mater. Photomicrographs, including scale, of the lesion site and micrometer‐based measurements of lesion dimension were obtained to establish the original lesion area. The scalp wound was then closed with interrupted 6‐0 nylon suture. For analgesia, Meloxicam (2 mg/kg SC) was administered before surgery, and then once per day for 2 days thereafter.

During the same surgical setting, after shaving the left or right flank of the animal anterior to the hip and then aseptically preparing the surgical area, a small skin incision was made with scissors, and a subcutaneous tunnel formed for insertion of an Alzet osmotic pump (Model 1002 14‐day infusion) containing either recombinant human relaxin (rhRLX) or vehicle (20 mmol/L sodium carbonate, pH 5.2). The skin was subsequently closed with 1 or 2 miniature auto‐clips.

After surgical recovery, the mice were placed into their home cages for ~11 weeks to allow for healing of cranial lesions prior to euthanasia, quantitation of lesion closure, and blood vessel parameters in the lesion area (see below). Approximately 8 days after the start of the infusion, ~100 *μ*L of blood was drawn from the tail vein into heparinized capillary tubes for preparation of plasma for measurement of rhRLX by ELISA (see below).

### Protocol 2 – recombinant human relaxin subcutaneous infusion: 0.05 *μ*g/h

In this protocol, the male mice were 12–13 weeks of age at the time of surgery. The percent lesion closure was comparably high in Protocol 1 regardless of vehicle or rhRLX administration; therefore, we made adjustments in Protocol 2 in two major ways. First, we euthanized the mice 10–12 days instead of 11 weeks after making the cranial lesions and implanting the osmotic pumps, and we reduced the rhRLX infusion rate to reach a lower circulating concentration than in Protocol 1. Otherwise procedures were the same as in Protocol 1.

### Protocol 3 – recombinant human relaxin subcutaneous infusion: 0.20 *μ*g/h

Male mice were 13–14 months of age at the time of surgery. The purpose of using older mice was to assess whether the rate of lesion closure might be retarded compared to younger mice, and consequently, more responsive to rhRLX treatment. In Protocol 3, we administered rhRLX by subcutaneous osmotic pump at a rate of 0.2 *μ*g/h, in order to reach plasma concentrations between those of Protocol 1 and 2. In addition, we euthanized the animals ~5 weeks after making the bilateral cranial lesions, which in this study were 3 mm in diameter. The purpose of making larger diameter lesions was to determine whether the degree of closure would be less extensive relative to the smaller diameter of ~1.5 mm, and consequently, more responsive to rhRLX treatment. Otherwise procedures were the same as in Protocol 1.

### Protocol 4 – recombinant human relaxin locally administered by collagen scaffolds: 1.0 *μ*g/scaffold

Male mice were 13–14 months of age at the time of surgery, in which a unilateral 3 mm cranial lesion was made. The amount of rhRLX impregnated into each scaffold (Bio‐Gide Geistlich Biomaterials, Princeton NJ) was based on an in vitro release assay (see below). The scaffolds containing rhRLX or vehicle were applied to the lesion, and then by applying a droplet or two of Vetbond Tissue Adhesive to the outer perimeter at several points, they were tacked to the bone. As in Protocol 3, we euthanized the mice ~5 weeks after surgery. Additionally, tail blood was obtained as previously described, in order to determine whether systemic levels of rhRLX would be detectable after local application by scaffold. Otherwise procedures were the same as in Protocol 1.

### Bone processing

In Protocol 1, mice were deeply anesthetized with isoflurane, and then after thoracotomy and heart puncture, 2–3 mL heparinized saline were injected into the ventricle followed by aspiration until the mouse was exsanguinated. The mouse was next perfused with 4% PFA, and the calvarium and the right femur (an uninjured long‐bone) harvested and placed in 4% PFA at 4°C for 24 h, washed three times with 1× PBS, and then stored in 70% ethanol for high‐resolution three‐dimensional (3D) microcomputed tomography (*μ*CT, see below). Following *μ*CT (Bouxsein et al. 2010), the parietal bones encompassing the bone defect lesions were decalcified, dehydrated in increasing concentrations of ethanol and embedded in paraffin. Paraffin blocks were sectioned at 5 *μ*m using an Accu‐Cut SRM 200 Sakura microtome (Sakura Finetek Europe B.V, Zoeterwoude, The Netherlands). Tissue sections were then stained with H&E, coverslipped, and examined under a microscope Olympus BX43 (Olympus Center Valley, PA). In addition, an histomorphometric evaluation of angiogenesis was performed on the bone defects sections by labeling blood vessels with a specific blood vessel marker using immunohistochemistry techniques (see below).

For all measurements, observers were blinded to treatment.

In Protocols 2–4, mice were deeply anesthetized by isoflurane and then decapitated. After dissecting and cleaning the calvarium, it was placed in 4% PFA at 4°C for 24 h, washed three times with 1× PBS, and then stored in 70% ethanol until analysis as described above.

### High‐resolution three‐dimensional (3D) microcomputed tomography (*μ*CT)

The parietal region of the calvaria and right femur were scanned ex vivo with a Bruker Skyscan 1172 *μ*CT (Kontich, Belgium) to determine the effects of rhRLX on lesion closure and bone morphometry remote from the injury site, respectively, using our protocols that abide by guidelines of the American Society of Bone and Mineral Research (Bouxsein et al. 2010; Beck et al. 2014; Beggs et al. 2015). Briefly, calvaria from Protocols 1–4 were scanned at 50kVP/200 *μ*A with a 0.5 mm aluminum filter, 2k camera resolution, 10 *μ*m voxel 0.7° rotation step, and 180° tomographic rotation. Cross‐sectional images were reconstructed using a filtered back‐projection algorithm (NRecon, Kontich, Belgium). A region of interest (ROI) that encompassed the entire lesion area was determined (CTAn, Konitch, Belgium) (Fig. S1A). Bone volume (BV, mm^3^) and bone volume fraction (BV/TV, %) were calculated within this ROI. Volumetric bone mineral density (BMD) and tissue mineral density (TMD) were also calculated, following calibration with hydroxyapatite phantoms. Models of parietal calvaria were generated in CTVox (Bruker, Kontich, Belgium) and images were exported to Image J (NIH) for 2D analysis of lesion closure, defined as bone area per original lesion area (%). Original lesion area was determined by assessing photomicrographs and micrometer‐based measurements that were acquired at surgery. Additionally, left and right parietal lesions within the same animal were examined across groups to verify reproducibility of the lesion area and closure parameters. For Protocol 4, two separate ROIs were developed that included bone within the lesion area and bone within the lesion area plus that surrounding the scaffolding area because the scaffolding extended into areas surrounding the original lesion (Fig. S1B).

Femurs (Protocol 1 only) were scanned in an identical manner at 7.2 *μ*m voxel. Images were reconstructed, as described above. The cancellous ROI at the distal femoral metaphysis began 0.75 mm proximal to the growth plate and encompassed 1 mm. The cortical ROI at the femoral diaphysis encompassed a 1 mm region at 45% of the femoral length, to avoid the third trochanter. 2D and 3D morphometric measurements at the distal femoral and femoral diaphysis ROIs included: cancellous bone volume (BV/TV, %), trabecular number (Tb.N, #/mm), trabecular separation (Tb.Sp, mm), trabecular thickness (Tb.Th, mm), trabecular pattern factor (Tb.Pf, #/mm), total cross‐sectional area inside the periosteal envelope (Tt.Ar, mm^2^), cortical bone area (Ct.Ar, mm^2^), medullary area (Ma.Ar, mm^2^), cortical area fraction (Ct.Ar/Tt.Ar, %), and 3D cortical thickness (Ct.Th, mm) (Bouxsein et al. 2010).

### Quantification of blood vessels in the calvarial defects

An evaluation of angiogenesis was performed during the healing phase of the calvarial defects. Blood vessel number (#/mm^2^), blood vessel area (%) and blood vessel perimeter (mm) were assessed in the area of the calvarial defects by labeling blood vessels in the histologic sections with a specific marker of blood vessels using immunohistochemistry techniques. For this purpose, paraffin‐embedded sections (5 *μ*m) of decalcified transverse sections of calvariae were deparaffinized and rehydrated through graded alcohols. Endogenous peroxidase was quenched by treatment with 3% hydrogen peroxide in methanol for 10 min. Heat‐mediated antigen retrieval was performed by incubating slides for 25 min at 96°C in DAKO Target Retrieval Solution pH 6.0 (Clara, CA). Sections were then blocked for 20 min in 10% goat serum in tris‐buffered saline with 1% Tween, rinsed, and blocked with Avidin/Biotin blocking kit (Vector Labs, Burlingame, CA) for 30 min. Slides were incubated overnight at 4°C in a humidified chamber with a purified rat anti‐mouse Pan Endothelial Cell antibody (MECA‐32; BD Pharmingen, Cat #550563) at a concentration of 1.56 *μ*g/mL in antibody diluent (Zymed Laboratories Inc). The secondary antibody was a biotinylated goat anti‐rat IgG antibody, mouse absorbed (1:100 dilution; Vector Laboratories Cat#BA‐9401). The antigen was visualized with Vectastain ABC Elite kit (Vector Laboratories, Burlingame, CA). Diaminobenzidene (DAB) was used as the chromogen. Negative controls in sections incubated without primary antibody demonstrated absence of signal. Slides were counterstained with hematoxylin (QS; Vector Laboratories), dehydrated, cleared in xylene, mounted in Permount (Fisher Scientific), and examined by light microscopy.

### In vitro rhRLX release assay

Bio‐Glide collagen sheets were cut into 3–4 mm diameter circular disks. This size completely absorbed 10 *μ*L fluid. Stock rhRLX was diluted accordingly with DPBS without calcium/magnesium containing penicillin/streptomycin and 0.1% BSA to yield 0.5 and 5.0 *μ*g rhRLX. After sterilization by autoclave, the small disks were suffused with 0.5 or 5.0 *μ*g rhRLX each in 10 *μ*L volume, and allowed to dry for 2 h. The disks containing rhRLX were next placed into wells of a 24‐well plate with 0.4 mL DPBS without calcium/magnesium containing penicillin/streptomycin and 0.1% BSA. The conditioned medium was harvested after 1, 2, 3, 4, 6, 8, and 10 days at 37°C in a cell culture incubator. Recombinant human relaxin was measured in the conditioned medium using the R&D ELISA as described below. Based on this in vitro release assay, a dose of 1.0 *μ*g rhRLX/scaffold was used in Protocol 4 above (see Results).

### Relaxin ELISA

rhRLX concentration in plasma or conditioned media was measured in duplicate using a commercially available human ELISA also validated for mouse plasma as a matrix (R&D Systems, Minneapolis, MN). The lowest standard in the assay was 7.8 pg/mL. According to the manufacturers specifications, the intra and interassay precision ranged from 2.3 to 4.7 and 5.5 to 10.2%, respectively (average CV of variation). Recovery after spike was 91–104% and linearity 105–111% of expected concentrations. As expected, plasma rhRLX was undetectable in vehicle‐treated mice (data not shown).

### Statistical analysis

Data were expressed as mean ± SE for each group. For Protocols 1–3, paired samples *t*‐tests were used to assess differences in initial lesion width, height, area, and volume, as well as closure for the bilateral (left vs. right) lesions in the same animal. Right and left bilateral lesion closures were also correlated. Because there were no differences between the left and right lesions (see Results), parameters were averaged within each animal, and compared between groups using independent samples t‐tests. Femur parameters in Protocol 1 and lesion values in Protocol 4 were compared between groups using independent samples t‐tests because bilateral values were not present for these outcomes. The blood vessel parameters were also compared between the relaxin and vehicle treatment groups using independent sample‐*t* tests. *P* values less than 0.05 were considered to be statistically significant.

## Results

Representative photographs of the cranial lesions are depicted in Figure S2. Overall, the baseline left and right lesion widths were 1.78 and 1.79 mm, respectively (*N* = 28, *P* = 0.947) with a correlation coefficient of 0.97 (*P* < 0.001). Similarly, the baseline left and right lesion heights were 1.91 and 1.94 mm, respectively (*N* = 28, *P* = 0.435) with a correlation coefficient of 0.95 (*P* < 0.001). Overall, the left and right lesion closures were also comparable – 63.7 and 61.3%, respectively (*P* = 0.401) with a correlation coefficient of 0.60 (*P* = 0.001). Left and right calvarial BMD, TMD, BV, and BV/TV were also not statistically significant from each other (*P* = 0.43–0.87; data not shown). Thus, we averaged the left and right lesion values for all subsequent analyses.

### Protocol 1 – recombinant human relaxin subcutaneous infusion: 1.0 *μ*g/h

There were no significant differences between rhRLX and vehicle treatments for lesion closure (%), bone volume (mm^3^), bone volume fraction (BV/TV%), bone mineral density (BMD g/cm^3^), and tissue mineral density (TMD g/cm^3^) (Table 1). Plasma rhRLX was 53 ± 9 ng/mL [range 33–89].

**Table 1:** Influence of relaxin (or vehicle) administration by subcutaneous osmotic pump on cranial lesion closure

	Lesion Closure (%)		BV(mm^3^)		BV/TV (%)		BMD (g/cm^3^)		TMD (g/cm^3^)	
	V	R	V	R	V	R	V	R	V	R
Protocol 1	73.5 ± 3.5	67.0 ± 3.4	0.172 ± 0.012	0.165 ± 0.012	35.3 ± 1.4	34.3 ± 1.8	0.441 ± 0.015	0.428 ± 0.021	0.973 ± 0.007	0.964 ± 0.008
*P*‐value	0.216		0.709		0.696		0.635		0.424	
Protocol 2	54.3 ± 2.2	45.8 ± 3.6	0.128 ± 0.011	0.104 ± 0.010	25.6 ± 0.9	21.9 ± 1.3	0.351 ± 0.009	0.310 ± 0.015	0.942 ± 0.006	0.943 ± 0.005
*P*‐value	0.072		0.143		0.039		0.039		0.903	
Protocol 3	70.3 ± 9.5	76.1 ± 5.1	0.440 ± 0.141	0.457 ± 0.024	28.3 ± 6.0	33.3 ± 2.6	0.371 ± 0.072	0.428 ± 0.025	1.001 ± 0.030	1.002 ± 0.004
*P*‐value	0.591		0.895		0.444		0.434		0.963	

Mean ± SEM. BV, bone volume; BV/TV, bone volume fraction; BMD, bone mineral density; TMD, tissue mineral density; V, vehicle; R, recombinant human relaxin.

Protocol 1: mice were euthanized ~11 weeks after implementing bilateral 1.5 mm cranial lesions and subcutaneous implantation of 14 day osmotic pumps containing recombinant human relaxin (rhRLX; 1.0 *μ*g/h) or vehicle (*n* = 6 mice each for relaxin and vehicle treatments).

Protocol 2: mice were euthanized 11–12 days after implementing bilateral 1.5 mm cranial lesions and subcutaneous implantation of 14 day osmotic pumps containing recombinant human relaxin (rhRLX; 0.05 *μ*g/h) or vehicle (*n* = 6 mice each for relaxin and vehicle treatments).

Protocol 3: mice were euthanized ~5 weeks after implementing bilateral 3.0 mm cranial lesions and subcutaneous implantation of 14 day osmotic pumps containing recombinant human relaxin (rhRLX; 0.2 *μ*g/h) or vehicle (*n* = 4 relaxin and *n* = 3 vehicle treated mice).

### Protocol 2 – recombinant human relaxin subcutaneous infusion: 0.05 *μ*g/h

Because rhRLX can have a biphasic dose response curve in vivo (Danielson and Conrad 2003; Debrah et al. 2005), we also explored a lower infusion rate that yielded an average circulating level of 1.5 ± 0.5 ng/mL [range 0.35–3.41]. As shown in Table 1, there were borderline significant or significant differences between rhRLX and vehicle treatments for lesion closure (vehicle 54.3 vs. relaxin 45.8%), BV/TV (vehicle 25.6 vs. relaxin 21.9%), and BMD (vehicle 0.351 vs. relaxin 0.310 g/cm^3^).

### Protocol 3 – recombinant human relaxin subcutaneous infusion: 0.20 *μ*g/h

An intermediate infusion rate produced a plasma rhRLX concentration of 4.9 ± 1.3 ng/mL [range 2.0–7.9]. However, rhRLX administration again failed to improve lesion closure or other bone parameters (Table 1).

### Protocol 4 – recombinant human relaxin locally administered by collagen scaffolds: 1.0 *μ*g/scaffold

Because systemic rhRLX administration was ineffective, we next tried local application by collagen scaffolds permeated with rhRLX. In order to establish a dose of rhRLX for delivery by the collagen scaffolds, we first performed an in vitro release assay (Wen et al. 2011; Charles et al. 2015). 0.5 and 5.0 *μ*g rhRLX were tested. The cumulative release over a period of 10 days was comparable between the doses (% of initial dose ~11.5%; Table 2). However, the rhRLX concentrations in the conditioned media differed markedly. For the 5.0 *μ*g dose, it ranged from 815 ng/mL on day 1 to 3.4 ng/mL on day 10. The concentrations for the 0.5 *μ*g dose were 77 and 0.4 ng/mL, respectively. Because the 5.0 *μ*g dose generally produced pharmacological concentrations especially in the first 2 days, and the 0.5 *μ*g dose yielded concentrations after 6 days that were generally low, we selected an intermediate dose of 1.0 *μ*g. The treatment was indeed locally confined, because circulating rhRLX was undetectable (below the lowest ELISA standard of 7.8 pg/mL). Once again, however, there were no significant differences between rhRLX and vehicle‐infused collagen scaffolds for percent lesion closure, BV, BV/TV, BMD, or TMD, whether bone in lesion only, or bone in lesion and scaffolding of surrounding area was analyzed for BV and TMD (Fig. S1A and B; Tables 3 and 4).

**Table 2:** In vitro release of recombinant human relaxin from Bio‐Gide collagen disks

	Days						
	1	2	3	4	6	8	10
Bio‐Gide Collagen containing:							
Relaxin 0.5 *μ*							
ng/mL	77.3	50.8	7.3	3.4	0.6	0.5	0.4
ng	30.9	20.3	2.9	1.4	0.25	0.18	0.15
Cumulative release (% initial dose)	6.2	10.2	10.8	11.1	11.2	11.2	11.2
Relaxin 5.0 *μ*							
ng/mL	815	508	57	29	5.8	4.3	3.4
ng	325.8	207.0	22.8	11.6	2.3	1.7	1.4
Cumulative release (% initial dose)	6.5	10.7	11.1	11.3	11.4	11.4	11.5

Recombinant human relaxin released from the collagen scaffolds was measured in the conditioned media for up to 10 days. See Methods for details.

**Table 3:** Influence of local relaxin (or vehicle) application by Bio‐Gide collagen scaffold on cranial lesion closure (lesion, only)

	Lesion closure (%)		BV‐1 (mm^3^)		BV/TV (%)		BMD‐1 (g/cm^3^)		TMD‐1 (g/cm^3^)	
	V	R	V	R	V	R	V	R	V	R
Protocol 4	81.3 ± 2.8	85.8 ± 3.3	0.899 ± 0.037	0.969 ± 0.122	34.7 ± 1.7	39.8 ± 2.3	0.451 ± 0.021	0.486 ± 0.027	1.013 ± 0.010	1.011 ± 0.009
*P*‐value	0.335		0.604		0.132		0.334		0.888	

Mean ± SEM. BV‐1, bone volume in lesion only; BMD‐1, bone mineral density in lesion only; TMD‐1, tissue mineral density of bone in lesion only; V, vehicle; R, recombinant human relaxin.

Protocol 4: mice were euthanized ~5 weeks after implementing 3.0 mm unilateral cranial lesions and applying scaffolds containing rhRLX (1.0 *μ*g/scaffold) or vehicle (*n* = 4 mice each for relaxin and vehicle treatments).

**Table 4:** Influence of local relaxin (or vehicle) application by Bio‐Gide collagen scaffold on cranial lesion closure (lesion and scaffolding)

	BV‐2 (mm^3^)		TMD‐2 (g/cm^3^)	
	V	R	V	R
Protocol 4	1.383 ± 0.126	1.515 ± 0.191	0.967 ± 0.019	0.964 ± 0.007
*P*‐value	0.586		0.899	

Mean ± SEM. BV‐2, bone volume in lesion and scaffolding of surrounding area; TMD‐2, tissue mineral density of bone in lesion and scaffolding of surrounding tissue; V, vehicle; R, recombinant human relaxin. Protocol 4: mice were euthanized ~5 weeks after implementing 3.0 mm unilateral cranial lesions and applying scaffolds containing rhRLX (1.0 *μ*g/scaffold) or vehicle (*n* = 4 mice each for relaxin and vehicle treatments).

### Influence of relaxin (or vehicle) administration by subcutaneous osmotic pump on cancellous and cortical morphometry of the femur

The femur was harvested in Protocol 1, in order to determine whether rhRLX might have effects on uninjured bone remote from the cranial lesion. Interestingly, trabecular thickness (mm), and trabecular pattern factor (#/mm) were significantly higher and lower, respectively, in the cancellous bone of the relaxin‐treated mice (both *P* < 0.05 vs. vehicle; Table 5). The bone volume fraction showed a borderline significant increase with relaxin administration (*P* = 0.094). There were no significant differences between relaxin and vehicle treatments in cortical bone morphometry (Table 5).

**Table 5:** Influence of relaxin (or vehicle) administration by subcutaneous osmotic pump on cancellous and cortical morphometry of the femur

	BV/TV (%)		Tb.Th (mm)		Tb.N (#/mm)		Tb.Sp (mm)		Tb.Pf (#/mm)	
	V	R	V	R	V	R	V	R	V	R
Cancellous morphometry										
Protocol 1	1.81 ± 0.19	2.74 ± 0.38	0.037 ± 0.001	0.044 ± 0.002	0.494 ± 0.049	0.626 ± 0.075	0.377 ± 0.030	0.379 ± 0.025	51.1 ± 0.65	42.8 ± 2.69
*P*‐value	0.094		0.030		0.229		0.957		0.040	
	3D Ct.Th (mm)		Tt.Ar (mm^2^)		Ct.Ar (mm^2^)		Ma.Ar (mm^2^)		Ct.Ar/Tt.Ar (%)	
	V	R	V	R	V	R	V	R	V	R
Cortical morphometry										
Protocol 1	0.194 ± 0.004	0.193 ± 0.002	1.554 ± 0.026	1.616 ± 0.048	0.705 ± 0.011	0.719 ± 0.010	0.849 ± 0.025	0.897 ± 0.041	0.454 ± 0.008	0.446 ± 0.009
*P*‐value	0.897		0.313		0.360		0.363		0.560	

Mean ± SEM. V, vehicle; R, recombinant human relaxin.

Protocol 1: mice were euthanized ~11 weeks after implementing bilateral 1.5 mm cranial lesions and subcutaneous implantation of 14 day osmotic pumps containing recombinant human relaxin (rhRLX; 1.0 *μ*g/h) or vehicle (*n* = 6 mice for relaxin and *n* = 4 mice for vehicle treatments). BV/TV, bone volume fraction; Tb.Th, trabecular thickness; Tb.N, trabecular number; Tb.Sp, trabecular separation; Tb.Pf, trabecular pattern factor; Ct.Th, cortical thickness; Tt.Ar, total area inside the periosteal envelope; Ct.Ar, cortical bone area; Ma.Ar, medullary area; Ct.Ar/Tt.Ar, cortical bone area fraction.

### Quantification of blood vessels in the calvarial defects

**Figure 2:**
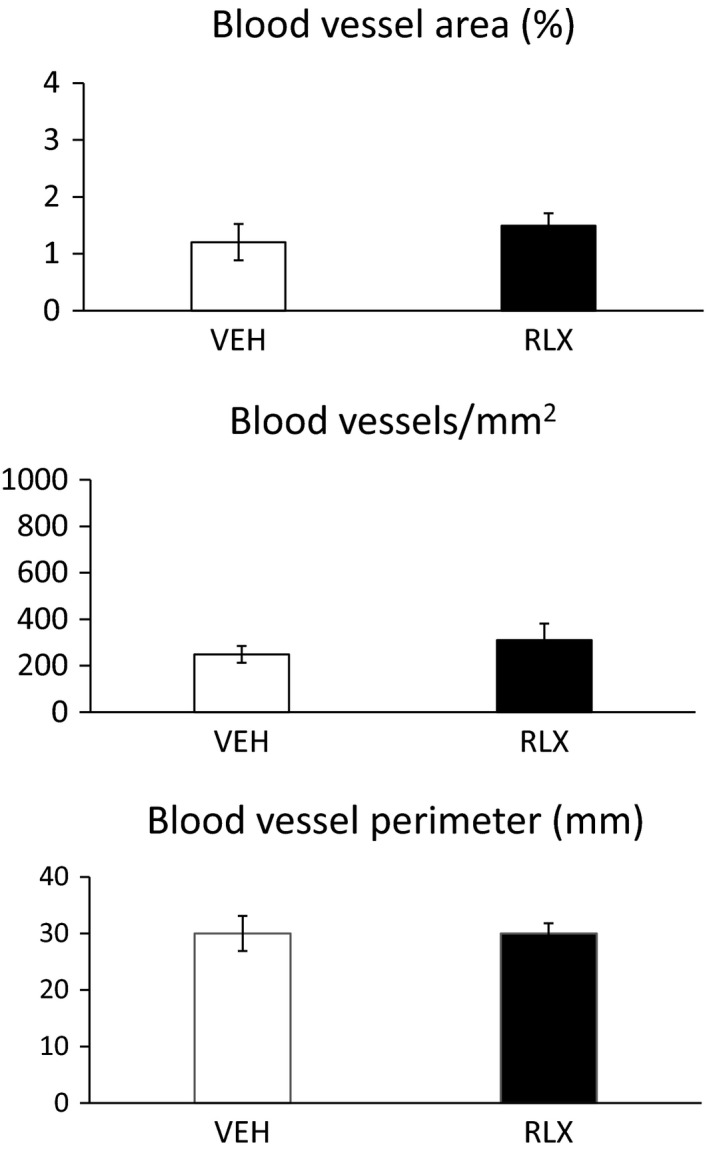
Protocol 1. Quantification of blood vessels in the calvarial defects.

**Figure 3:**
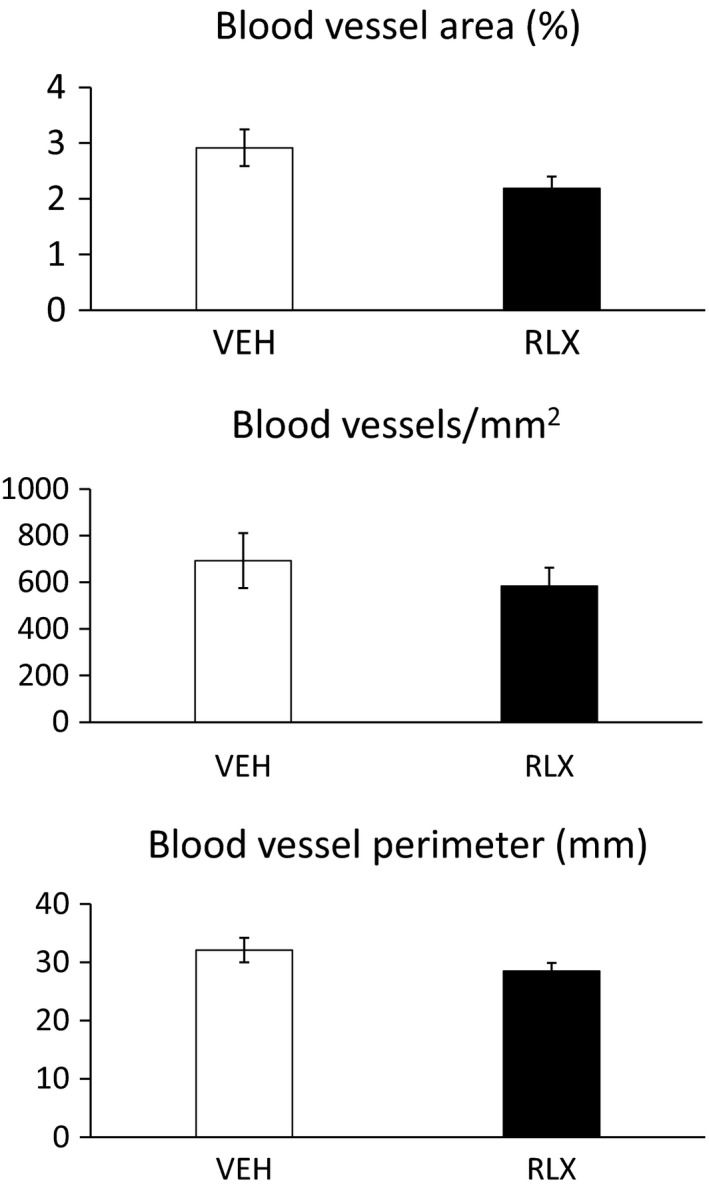
Protocol 2. Quantification of blood vessels in the calvarial defects.

No differences were observed in blood vessel area (%), blood vessel number (#/mm^2^), and blood vessel perimeter (mm) in the region of the calvarial defects between mice administered vehicle or relaxin for 2 weeks starting at the time of lesioning, and then euthanized 11 weeks later (Protocol 1; Fig. 2). Similarly, no differences were observed in these parameters between vehicle and relaxin administered mice that were euthanized 10–12 days after lesioning and start of the vehicle or relaxin administration (Protocol 2; Fig. 3). However, independent of the treatment group, mice at 10–12 days postlesioning (protocol 2) had greater blood vessel area (%) and blood vessel number (#/mm^2^), but not blood vessel perimeter (mm) compared to mice after 11 weeks (protocol 1; compare Figs. 2 and 3).

## Discussion

A major impediment to bone healing is the disruption of osseous blood flow. Hence, therapeutic agents that will improve bone perfusion may accelerate healing of fracture lesions. To this end, we reasoned that relaxin may potentially be a therapeutic molecule, because the hormone possesses biological attributes that theoretically speaking could improve (1) local angiogenesis, (2) vasculogenesis and osteogenesis by mobilizing and activating BMPC, (3) blood flow via vasodilation, (4) bone formation by directly acting on osteoblasts and/or osteoclasts, and reduce (5) inflammation (Masini et al. 2004; Jeyabalan et al. 2007; Conrad 2010, 2011; Brecht et al. 2011; Segal et al. 2012; Ferlin et al. 2017). Moreover, relaxin has a good safely record based on human clinical trials for other indications (Said and Mukherjee 2013; Ghosh et al. 2017).

Our preclinical study in mice did not support a beneficial role for relaxin in bone lesion healing whether administered systemically or locally (Tables 1, 3, and 4). Despite implementing a rhRLX dose response reaching plasma concentrations which spanned the physiological to supraphysiological range with mean levels of 1.5, 4.6, and 53 ng/mL; testing two different sizes of calvarial lesions (~1.5 and ~3.0 mm); imposing different intervals of time from imposition of bone lesions and treatments to euthanasia (1.6, 5, and 11 weeks); and investigating different mouse ages (3–4 and 13–14 months), we were unable to elicit a salutary role for relaxin in bone lesion healing. Consistent with the lack of accelerating calvarial defect closure, there was also an absence of any beneficial effect on vascularity in the bone lesions by relaxin. One possible explanation for lack of therapeutic effect is that the relaxin receptor, RXFP1, is not expressed in all blood vessels, and perhaps those relevant to perfusion necessary for healing of the calvaria are devoid of receptors (Jelinic et al. 2014). To our knowledge, RXFP1 expression has not been measured in these blood vessels, although parenchymal and middle cerebral arteries were reported to be devoid of RFXP1 and RXFP2 in the rat as determined by reverse transcription‐quantitative polymerase chain reaction even after RNA amplification (Chan et al. 2013). Speculating further, calvarial osteoblasts and/or osteoclasts may also be devoid of RXFP1 receptors, thus precluding a direct action of relaxin on bone formation. Nevertheless, independently of the experimental group, the angiogenic process was more active at the earlier time point after implementing the cranial bone lesion (10–12 days), than after 11 weeks in support of bone healing (Figs. 2, 3 and 3).

Unexpectedly, relaxin diminished several cranial bone healing parameters by ~12–16% for lesion closure (%), BV/TV(%), and BMD (g/cm^3^) in Protocol 2 – differences which were on the threshold of being biologically significant (Table 1). However, Protocol 2 was the shortest duration (mice euthanized 11–12 days postlesioning) perhaps suggesting that relaxin may have slightly delayed calvarial lesion closure shortly after implementation. However, the longer duration Protocols did not support these observations providing no evidence that relaxin delayed closure in studies lasting 5–11 weeks.

A somewhat incidental finding was that relaxin may have stimulated trabecular bone growth in an uninjured bone (femur) as evidenced by higher Tb.Th and lower Tb.Pf (Table 5). This finding is consistent with a previous report that relaxin may stimulate osteoblast activity, resulting in bone formation (Ferlin et al. 2017). The results support the possibility that, perhaps in contrast to the calvaria, femur vasculature, osteoblasts and/or osteoclasts may express RXFP1 receptors, thus leading to enhanced trabecular growth by relaxin. However, a larger study utilizing histomorphometry is necessary to confirm whether relaxin may have produced a bone anabolic or antiresorptive effect in the cancellous bone of the femur. It is possible that relaxin may have an effect in skeletal bones with endochondral ossification, but not in craniofacial bones with intramembranous ossification and periosteal bone formation.

Significant strengths of this work include the overall study design, which was carefully and deliberately constructed; state‐of‐the‐art *μ*CT approaches for quantifying bone lesion closure volume and density; and implementation of appropriate protocol changes as dictated by the results as they emerged throughout the study. Potential weaknesses include the fact that we did not implement all possible combinations of the protocol changes, and there was a limited number of mice per group. Even though for the majority of experimental protocols, we met the power analysis criteria based on Wang and colleagues (Wang et al. 2011), this work should still be considered a pilot study. Another possible drawback is that we had sufficient funding to investigate only one sex. We chose males, because to date, the majority of front‐line combatants at risk for bone trauma are men. However, we have not observed any differences in the physiological responses of males and females to relaxin administration at least in the cardiovascular system, which may have bearing on the present work that mainly was focused on osseous blood supply (Danielson et al. 2000; Debrah et al. 2005). Nevertheless, future investigations definitely need to include female animals. Perhaps relaxin would prove beneficial in repairing calvarial lesions in females.

In summary, relaxin did not accelerate calvarial lesion closure in a mouse model. However, this study should not dissuade future investigation of a potential salutary role for relaxin by testing different animal species and experimental models of bone fracture, as well as the female sex.

## Conflict of Interest

KPC discloses use patents for relaxin. The other authors have declared that no conflict of interest exists.

## Supporting information




**Figure S1.** (A) Bilateral lesion ROI. (B) Bone area only and Bone +Scaffold ROI bone area only ROI.

**Figure S2.** Representative photomicrographs of bilateral cranial lesions.
