# Supplementary figures and images for: Potential therapeutic use of relaxin in accelerating closure of cranial bone defects in mice
Source: Physiol Rep. 2019 Jun 2;7(11):e14106. doi: 10.14814/phy2.14106 (PMC6545299; doi:10.14814/phy2.14106)

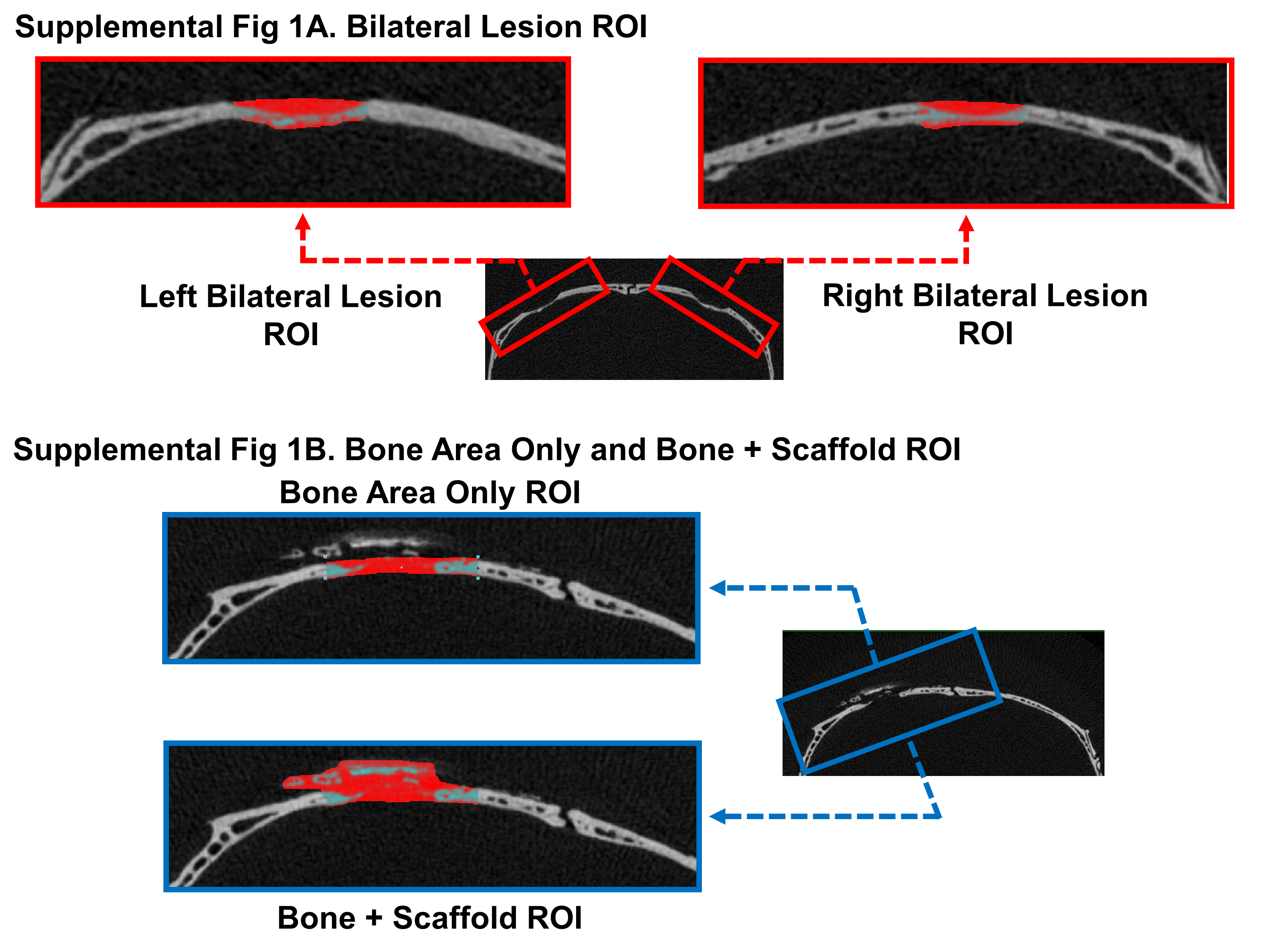

Supplement: Supplementary file 1 — Figure S1. (A) Bilateral lesion ROI. (B) Bone area only and Bone +Scaffold ROI bone area only ROI. [file PHY2-7-e14106-s001.tif]

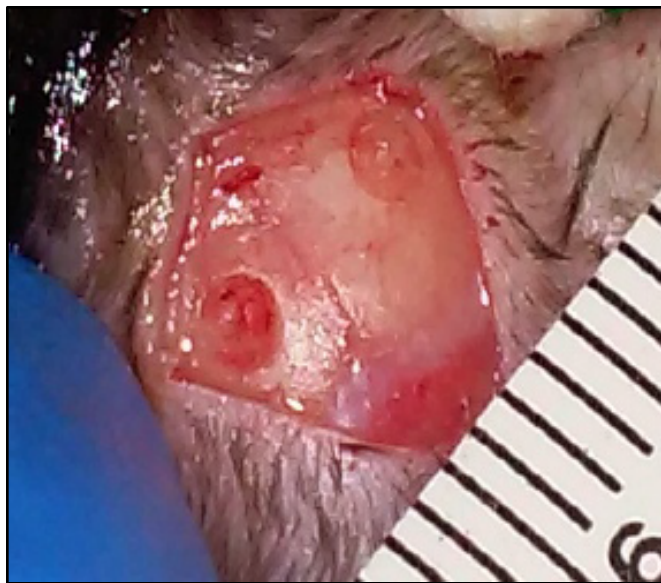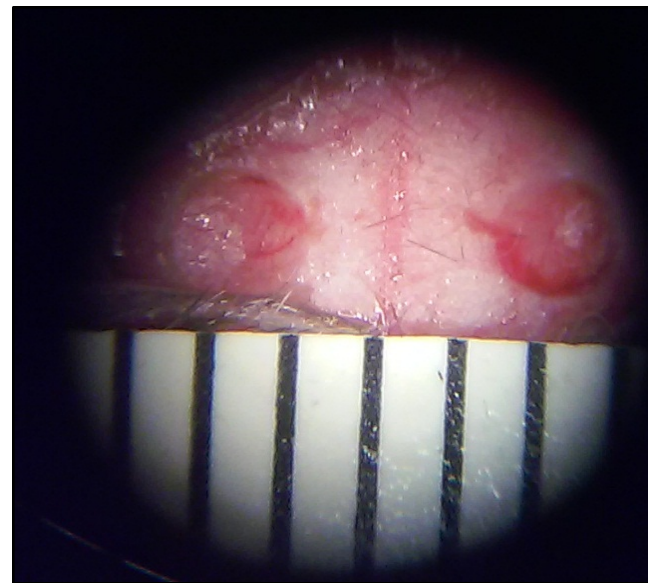

**Figure S2.** Representative Photomicrographs of Bilateral Cranial Lesions

Supplement: Supplementary file 2 — Figure S2. Representative photomicrographs of bilateral cranial lesions. [file PHY2-7-e14106-s002.pdf]
